# Relationships between Morning Thirst and Later Hydration Status and Total Water Intake

**DOI:** 10.3390/nu16183212

**Published:** 2024-09-23

**Authors:** Kelly B. Elliott, Marcos S. Keefe, Jan-Joseph S. Rolloque, Nigel C. Jiwan, Ryan A. Dunn, Hui-Ying Luk, Yasuki Sekiguchi

**Affiliations:** 1 Sports Performance Laboratory, Department of Kinesiology & Sport Management, Texas Tech University, Lubbock, TX 79409, USA; kelly.elliott@ttu.edu (K.B.E.); makeefe@ttu.edu (M.S.K.); jarolloq@ttu.edu (J.-J.S.R.); dunn77277@ttu.edu (R.A.D.); 2 Applied Physiology Laboratory, Department of Kinesiology & Sport Management, Texas Tech University, Lubbock, TX 79409, USA; jiwan@hope.edu (N.C.J.); huiying.luk@ttu.edu (H.-Y.L.)

**Keywords:** drinking behavior, fluid intake, thirst sensation, body fluid balance

## Abstract

*Purpose:* To investigate the effects of thirst on later hydration status, total water intake (TWI-MA), and its potential sex differences. *Methods:* Twelve men (mean ± standard deviation; age: 21 ± 2 years; mass: 81.0 ± 15.9 kg) and twelve women (age: 22 ± 3 years; mass: 68.8 ± 15.2 kg) visited the laboratory in the morning (first thing in the morning) and afternoon (2:00–4:00 p.m.) for three consecutive days under a free-living condition. At each visit, urine osmolality (U_OSM_), urine specific gravity (USG), urine color (U_COL_), body mass loss (BML), thirst, and plasma osmolality (P_OSM_) were collected and analyzed. The participants recorded their food and fluid intake between the visits to determine TWI-MA. Linear regression was used to predict the effect of morning thirst on the afternoon hydration indices for all the participants, as well as for males and females separately. *Results:* Higher morning thirst predicted lower U_OSM_ (r^2^ = 0.056, *p* = 0.045), USG (r^2^ = 0.096, *p* = 0.008), U_COL_ (r^2^ = 0.074, *p* = 0.021), and higher thirst (r^2^ = 0.074, *p* = 0.021) in the afternoon. However, morning thirst did not predict afternoon BML, P_OSM_, or TWI-MA (*p* > 0.05). In males, higher morning thirst predicted lower afternoon U_OSM_ (r^2^ = 0.130, *p* = 0.031) and USG (r^2^ = 0.153, *p* = 0.018). Additionally, higher morning thirst predicted higher TWI-MA (r^2^ = 0.154, *p* = 0.018) in females. *Conclusions:* Morning thirst had a negligible impact on later hydration status, specifically with afternoon urine indices. Furthermore, higher thirst sensation did not impact BML, P_OSM_, or TWI-MA. However, thirst sensation minimally contributed to drinking behavior in females. Overall, individuals may not rely solely on thirst sensation to manipulate their drinking behavior to optimize their fluid balance during their daily lives due to the complexity of thirst mechanisms.

## 1. Introduction

Water comprises 55–65% of the human body and is essential for the proper functioning of metabolism, temperature regulation, cellular homeostasis, and overall health [1,2]. Many major systems in the body, such as the cardiovascular, respiratory, gastrointestinal, and renal, require adequate water to operate, thus it is vital to maintain body fluid balance [3,4]. The body loses water through the skin, lungs, and kidneys from daily processes, such as sweating, breathing, and urinating [1]. In the United States, it is recommended that males and females consume approximately 3.4 and 2.6 L∙d^−1^ to achieve the sufficient fluid intake to maintain optimal hydration status, respectively [2]. However, it is common among all age groups to become dehydrated if fluid loss is not replenished [1,5]. Therefore, the body attempts to mitigate dehydration through the motivational stimulus thirst, which encourages individuals to drink fluids in an effort to maintain their body fluid balance and meet their hydration needs [5,6]. However, when individuals experience an increased thirst perception, the body is already experiencing a 1–2% body mass loss, which can indicate mild dehydration [7].

Thirst is an automatic response to physiological changes in osmotic content, which also stimulates the hormonal release of arginine vasopressin (AVP) to increase fluid retention [8,9]. More specifically, the oropharyngeal region plays a significant role in thirst sensation and AVP secretion, primarily through the dryness of the region, which is sensitive to increases in plasma osmolality (P_OSM_) [10]. Oropharyngeal receptors in the region closely monitor the volume and rate of fluid consumption and, through stimulation, can create a reflex inhibition of perceived thirst and AVP secretion, known as oropharyngeal metering [10]. Within the initial minutes of water ingestion, this oropharyngeal metering inhibition reduces the urge to drink (thirst sensation), which may potentially decrease the drinking rate [10]. As a result, this could impact the total water intake, referring to the amount of water ingested from foods and fluid and overall rehydration [2,10]. For example, thirst sensation and AVP levels can be reduced without changing the body fluid balance and the hydration status after drinking behavior [10]. In this situation, the body may misinterpret its actual hydration status, and fluid consumption might not be enough to maintain the fluid balance due to the lack of thirst, thus causing “voluntary dehydration” [8]. 

The sensation of thirst is interrelated with drinking behavior, as its primary role is to preserve homeostasis and replenish the accumulated water loss [7,11]. Moreover, drinking behavior is closely linked with thirst sensation as their relationship can have acute (i.e., within a day) and prolonged (i.e., the following day) effects. However, individuals sometimes do not respond to the thirst sensation by initiating their drinking behavior due to several factors, such as the time of day, their eating schedule, their gastric contents, their social interactions, and their fluid characteristics [7]. As a result, the previous literature found that only 2% of the time, people would consume fluids based on thirst (i.e., they drank fluids when thirsty), and approximately 62% of the time, their drinking behavior was not based on thirst (i.e., they were thirsty and did not consume fluids or they were not thirsty and consumed fluids) [6]. Therefore, people’s fluid consumption and drinking behavior may often occur more spontaneously, rather than people always responding to the thirst sensation to maintain their body fluid balance. 

Collectively, thirst perception might not be associated with changes in hydration status due to the nature of its complexity and human behavior [7,10]. However, the impact of thirst on total water intake and its relationship with changes in hydration status remains unclear [9]. Understanding the role of thirst perception in daily life is critical to optimizing body fluid balance. Therefore, the purpose of this study was to investigate the effects of thirst on later hydration status and total water intake in healthy individuals under a free-living condition, both overall and between males and females. Based on the aforementioned information regarding the relationship between thirst perception and its impact on drinking behavior, it is hypothesized that thirst might not significantly affect later hydration status. 

## 2. Methods

### 2.1. Participants

Twelve men (mean [M] ± standard deviation [SD]; age: 21 ± 2 years; mass: 81.0 ± 15.9 kg) and twelve women (age: 22 ± 3 years; mass: 68.8 ± 15.2 kg) volunteered to participate in the study. A power analysis with G*Power 3.1.9.7 (Universitat Kiel, Kiel, Germany) was conducted to determine that a total of twenty-four participants were needed for the study for a power of 0.8, an alpha level of 0.05, with an effect size of 0.2 [12]. Only women taking oral contraceptive pills participated and completed the study visits over their 7-day placebo pill week. All participants signed an informed consent document, completed a medical screening questionnaire, and reported not having kidney disease or a urinary tract infection at the time of the study. This study was approved by the University Institutional Review Board for human subject research and adhered to the Declaration of Helsinki.

### 2.2. Procedures

Participants visited the laboratory in the morning and the afternoon for three consecutive days under free-living conditions. Before all the morning visits, the participants were instructed to abstain from food or fluid consumption and not to perform any physical activity, in order to capture their measurements first thing in the morning. At each visit, a urine spot sample, a nude body mass measurement, their thirst level, and a venous blood sample were acquired. After the morning visit, the participants were instructed to consume food and fluids as they normally do in their free living. Then, the participants were asked to return to the laboratory between 2:00–4:00 p.m. to perform the same measurement procedures as their morning visit. The participants were also instructed to record their food and fluid intake between the morning and the afternoon visit to calculate their daily total water intake (TWI-MA) and caloric intake (KCAL) (ASA24, National Institute of Health, Bethesda, MD, USA). TWI-MA refers to the amount of water consumed from food and fluid intake between the morning and the afternoon visits. In addition to this, the participants reported to the laboratory (first thing in the morning) for three consecutive days in a euhydrated state (urine specific gravity [USG] < 1.020) after completing the free-living condition to establish their baseline body mass (BM) [13,14]. For these euhydrated laboratory visits, the participants were advised to consume additional amounts of water throughout the day and the night before their visit to ensure they arrived in a euhydrated state. 

### 2.3. Measurements

Each visit consisted of the collection of hydration makers, including the BM, urine indices, and thirst levels. The nude BM was recorded via an electronic scale (Health-o-Meter). The baseline BM was calculated by the average of the three consecutive euhydrated BM measurements that were taken first thing in the morning [13]. Then, the body mass loss (BML) was calculated based on baseline BM with the following equation: (Baseline BM − Each BM measurement)/Baseline BM × 100. Urine was utilized to assess the USG, using a handheld refractometer (ATAGO, Tokyo, Japan); the urine color (U_COL_), using a validated 8-point chart [15]; and the urine osmolality (U_OSM_), using an Advanced Instruments Osmometer Pro (Norwood, MA, USA). A blood sample was also collected to analyze the plasma osmolality (P_OSM_). Additionally, thirst level was evaluated with a Likert-type scale of 1 to 9, with 1 being “not thirsty at all” and 9 being “very, very thirsty” [16].

### 2.4. Statistical Analysis

A repeated-measures ANOVA was used to determine the difference between hydration markers in the morning versus the afternoon. Pearson’s product–moment correlation was used to calculate the relationship between TWI-MA and the hydration markers. Linear regression was used to predict the hydration indices in the afternoon from the morning thirst sensation. Also, a stepwise linear regression predicted thirst in the afternoon from the hydration indices in the morning. Pearson’s product–moment correlation, linear regression, and a stepwise linear regression were conducted for all the participants, as well as for males and females separately. These analyses were repeated after separating all the participants by their hydration status (euhydrated or dehydrated). KCAL was also analyzed using a repeated-measures ANOVA to compare sex differences (males and females). Pearson’s product–moment correlation was performed to determine associations between KCAL and all the variables for all the participants and also when categorized by their hydration status (euhydrated or dehydrated). Additionally, a two-way repeated-measures ANOVA was performed to investigate the differences in hydration markers between the sexes (males and females) and the time points (morning and afternoon). Lastly, a two-way repeated-measures ANOVA was performed to compare potential differences in the daily TWI-MA between males and females. Effect size (ES) was calculated using Cohen’s d, with the thresholds being small (0.2), medium (0.5), or large (≥0.8) effects [17]. The data are reported as M ± SD [95% confidence intervals (CI)] and ES. All the statistical analyses were completed using SPSS (Version 29, IBM Corp., Armonk, NY, USA), with the significance set at *p* < 0.05. 

## 3. Results

Morning BM (74.9 ± 16.3 kg) was lower than afternoon BM (75.1 ± 16.2 kg [−0.4, −0.1], ES = 0.01, *p* = 0.006), and morning BML (0.2 ± 0.9%) was greater than afternoon BML (−0.2 ± 0.9% [0.1, 0.5], ES = 0.4, *p* = 0.005). Morning thirst perception (5 ± 2) was significantly higher than afternoon thirst perception (3 ± 1 [1.6, 2.9], *p* < 0.001) with large effects (ES = 1.3). Morning U_COL_ (5 ± 1) was higher when compared to afternoon U_COL_ (4 ± 2 [0.6, 1.6], *p* < 0.001) with medium effects (ES = 0.6). Afternoon USG (1.014 ± 0.005) was significantly lower compared to morning USG (1.019 ± 0.005 [0.003, 0.007], ES = 1.0, *p* < 0.001). Also, afternoon U_OSM_ (589 ± 242 mmol·kg^−1^) was lower than morning U_OSM_ (690 ± 211 mmol·kg^−1^ [24.7, 176.1], *p* = 0.012) with small effects (ES = 0.4). Morning P_OSM_ (288 ± 3 mmol·kg^−1^) was significant when compared to afternoon P_OSM_ (286 ± 4 mmol·kg^−1^ [0.26, 3.48], ES = 0.6, *p* = 0.025). The average TWI-MA for all participants during the free-living days was (1467 ± 748 mL). 

Higher TWI-MA was associated with lower BML (r = 0.297, *p* = 0.011) and U_COL_ (r = 0.336, *p* = 0.004) in the afternoon. However, TWI-MA was not associated with any variables in the morning or the remaining afternoon variables, including U_OSM_ and USG (*p* > 0.05). Table 1 demonstrates the prediction of morning thirst on afternoon hydration markers. Higher morning thirst significantly predicted lower U_COL_ (r^2^ = 0.074, *p* = 0.021), USG (r^2^ = 0.096, *p* = 0.008), U_OSM_ (r^2^ = 0.056, *p* = 0.045), and higher thirst (r^2^ = 0.074, *p* = 0.021) in the afternoon. However, morning thirst did not predict BML, P_OSM_, and TWI-MA in the afternoon (*p* > 0.05). Increased thirst and BML in the morning together significantly predicted higher thirst (r^2^ = 0.125, *p* = 0.010) in the afternoon.

Table 2 examines the relationship between the morning and afternoon hydration indices, independent of thirst. Morning U_OSM_ was significantly associated with afternoon U_OSM_ (*p* < 0.001), USG (*p* = 0.001), U_COL_ (*p* = 0.008), and P_OSM_ (*p* = 0.005). Morning USG also had a significant relationship with the afternoon variables, such as U_OSM_ (*p* = 0.002), USG (*p* < 0.001), U_COL_ (*p* = 0.029), and P_OSM_ (*p* = 0.045). Additionally, morning U_COL_ was associated with afternoon U_OSM_ (*p* = 0.017), USG (*p* = 0.017), and U_COL_ (*p* = 0.022). Morning BML demonstrated a significant relationship with afternoon U_OSM_ (*p* = 0.001), USG (*p* < 0.001), U_COL_ (*p* = 0.001), and BML (*p* < 0.001). Morning BM was associated with afternoon BM (*p* < 0.001) and P_OSM_ (*p* = 0.028). There was no significant relationship between morning P_OSM_ and any of the afternoon variables (*p* > 0.05). Moreover, TWI-MA did not have a significant relationship with any of the morning variables (*p* > 0.05). 

When comparing the hydration markers between the sexes and the time points, there were no significant differences across all the variables (*p* > 0.05), as demonstrated by Table 3. 

There were no significant differences in the average daily TWI-MA between males (1516 ± 750 mL) and females (1418 ± 753 mL [−685, 489], ES = 0.13, *p* = 0.733). In males, higher TWI-MA was only associated with lower BML in the afternoon (r = 0.480, *p* = 0.003). However, higher TWI-MA in females was associated with higher morning thirst (r = 0.392, *p* = 0.018) and lower afternoon U_COL_ (r = 0.471, *p* = 0.004) and U_OSM_ (r = 0.342, *p* = 0.041). The remaining hydration markers were not associated with TWI-MA for both males and females (*p* > 0.05). Furthermore, in males, higher morning thirst predicted lower afternoon USG (r^2^ = 0.153, *p* = 0.018) and U_OSM_ (r^2^ = 0.130, *p* = 0.031). Conversely, higher morning thirst predicted higher TWI-MA (r^2^ = 0.154, *p* = 0.018) in females. All the other afternoon hydration indices were not predicted by morning thirst sensation for both sexes (*p* > 0.05). Lastly, in males, no morning hydration indices predicted thirst in the afternoon (*p* > 0.05). Despite this, higher morning BML predicted higher afternoon thirst perception (r^2^ = 0.184, *p* = 0.009) in females. 

Table 4 demonstrates the amount of TWI-MA between the sexes (males and females) and for all the participants for each time point (morning and afternoon), based on the USG levels being categorized into euhydrated (USG < 1.020), dehydrated (USG ≥ 1.020), and severely dehydrated (USG ≥ 1.030) [18]. Furthermore, all the participants were then separated into euhydrated (USG < 1.020) or dehydrated (USG ≥ 1.020) groups. In the dehydrated group, higher morning thirst predicted lower afternoon U_COL_ (r^2^ = 0.146, *p* = 0.026) and USG (r^2^ = 0.158, *p* = 0.020). However, TWI-MA was not associated with any variables at either time point (morning or afternoon) in the dehydrated group (*p* > 0.05). Also, morning hydration markers did not predict afternoon thirst in the dehydrated group (*p* > 0.05). On the contrary, higher morning thirst predicted higher afternoon thirst (r^2^ = 0.106, *p* = 0.046) in the euhydrated group. A stepwise linear regression demonstrated that higher morning BML predicted higher afternoon thirst (r^2^ = 0.202, *p* = 0.005) in the euhydrated group. Lastly, higher TWI-MA was associated with lower afternoon BML (r = 0.402, *p* = 0.012), U_COL_ (r = 0.512, *p* = 0.001), USG (r = 0.351, *p* = 0.031) and U_OSM_ (r = 0.458, *p* = 0.004) in the euhydrated group. 

The average KCAL was determined for all the participants (795 ± 518 kcal), for males (750 ± 550 kcal), and for females (840 ± 488 kcal), as well as for the participants who were separated by their hydration status being euhydrated (686 ± 477 kcal) or dehydrated (917 ± 542 kcal). For all the participants, higher morning USG (r = 0.253, *p* = 0.032), afternoon USG (r = 0.287, *p* = 0.018), and U_OSM_ (r = 0.246, *p* = 0.037) were associated with higher KCAL. Despite this, when comparing males and females, there were no differences between KCAL (*p* = 0.582). Similarly, there was no relationship between KCAL and any of the hydration makers when separating participants into euhydrated and dehydrated groups (*p* > 0.05). 

## 4. Discussion

The present study investigated the association of morning thirst sensation with later hydration status and TWI-MA. Our findings indicate that morning thirst did not predict afternoon P_OSM_, although it minimally contributed to the prediction of the afternoon urine indices. In general, morning thirst sensation demonstrated no relationship with later TWI-MA; however, thirst sensation did have a trivial impact on TWI-MA in females compared to males. Collectively, these results highlight that morning thirst sensation likely has a negligible impact on TWI-MA and on later hydration status. As such, morning thirst sensation may not influence individual drinking behavior for the remainder of the day. 

One of the factors that likely contributed to our present findings is the complexity of thirst sensation. For instance, the inhibitory mechanism of the oropharyngeal reflex plays a direct role in dissipating thirst sensation [10], which may suggest why morning thirst demonstrated a minimal impact on later TWI-MA. As a result, individuals are potentially less likely to increase their fluid consumption after an initial ingestion because their body may inaccurately assume that it has been sufficiently rehydrated [8]. In accordance with this, there is a rapid decrease in thirst sensation within the first 5 min of fluid consumption, despite the body fluid deficit not being fully restored [10,19]. Consequently, individuals may have ceased or reduced their overall fluid intake after the dissipation of the initial thirst sensation. In fact, Armstrong and colleagues (2020) indicated that approximately 65% of water intake was ingested during the first 2.5 min of drinking; following this initial drinking, there was a prompt decrease in the fluid consumption rate and the subsequent fluid volume [9]. This may contribute to the lack of influence shown in the present study regarding the effect of morning thirst sensation on subsequent TWI-MA. 

In line with these findings, morning thirst sensation also demonstrated little influence on later hydration status. It was previously identified that thirst perception was unchanged regardless of hydration status (euhydrated vs. dehydrated) after individuals consumed fluid under an ad libitum condition [19]. Furthermore, prior research has compared hydration biomarkers between low and high water consumers and determined that regardless of the water intake, their P_OSM_ and thirst sensation were not different across groups [7,20]. Similarly, we also observed suppressed thirst sensation in the afternoon, despite the urine indices, P_OSM_, and BML showing negligible differences in hydration status between the morning and afternoon measurements. Taken together, thirst sensation may be diminished following an initial fluid intake, regardless of the person’s hydration status. Therefore, the changes in thirst sensation do not necessarily correlate to changes in hydration status from fluid intake.

In addition to these physiological mechanisms, morning thirst perception potentially did not impact later TWI-MA and drinking behavior due to the elaborate neural network that promotes or diminishes thirst sensation [7,9,21,22,23]. More specifically, one of the most contributing factors influencing drinking behavior is the interrelationship between thirst and hunger sensations [24]. For example, Burnett et al. (2016) determined that neurons trigger hunger sensations to attenuate motivational sensations such as thirst and social behavior in mice [25]. Also, it was suggested that humans follow a similar hierarchy with these corresponding variables, as hunger sensations presumably affect perceived thirst and drinking behavior [24]. Thus, strong hunger sensations may alter fluid intake during meals, which could impact overall total water intake [24]. In fact, Castro (1988) determined that only 22% of fluid consumption occurred independent of food intake and accounted for approximately one-eighth of daily total water intake [23]. This indicates that the amount of fluid consumption is minimal outside of mealtimes when drinking behavior is initiated based on thirst sensation alone. In addition, McKiernan and colleagues (2008) found approximately 75% of total fluid intake corresponded with eating when monitoring fluid intake under a free-living condition [6]. Therefore, hunger sensations and food consumption have demonstrated a greater influence on the amount of fluid consumption, rather than thirst perception alone [23]. Together, these may explain why the current study observed no relationship between thirst and TWI-MA, as stronger stimuli, such as hunger sensations, could impact TWI-MA. 

In regard to sex, there was no significant differences in the morning and afternoon hydration markers and TWI-MA between males and females. Adams and colleagues (2020) demonstrated similar findings of no significant differences between males and females when examining 24 h urinary hydration markers [26]. Amongst males and females, there are differences in sex hormones which could contribute to AVP synthesis and osmoregulation [27,28,29]. Due to this, Stachenfeld et al. (2008) investigated the effects of sex hormones (i.e., estrogen and progesterone) and their effects on body fluid distribution, osmoregulation, AVP, and thirst in females [30]. They determined that sex hormones can cause slight shifts in osmoregulation; however, this change had a minimal impact on body fluid distribution as it mainly impacted extracellular fluid space [30]. Thus, there was practically no impact on overall body fluid balance, which could explain why the current study found no difference in hydration status between males and females. On the contrary, our study found that higher morning thirst predicted higher TWI-MA in females to a small degree. In this case, thirst sensation in females could respond differently than in males to changes in plasma osmolality, as sex hormones fluctuate throughout the menstrual cycle [31,32]. Additionally, previous studies have further indicated that a specific threshold for general plasma osmolality cannot be established because of how differently males and females perceive thirst sensation [31,32]. However, as formerly mentioned, the effect of thirst sensation on drinking behavior is highly complex, which is why it is difficult to determine what specific factors influence drinking behavior in females. Therefore, future studies should examine and identify what aspects may cause females to respond to thirst sensations slightly more than males. Altogether, females may minimally rely more on thirst sensation to initiate drinking behavior than males, despite their overall TWI-MA being similar throughout the day. 

There are some limitations to the current study that could have potentially impacted the results. First, we did not directly measure the oropharyngeal reflex or AVP secretion; hence, we could not precisely determine the impact of these factors on thirst sensation. However, based on the previously mentioned relationship between thirst, the oropharyngeal reflex, and AVP, these factors likely played a role in thirst sensation [10]. Second, there was an absence of a hunger sensation questionnaire, which could have provided further data to support its influence in TWI-MA. Although hunger sensation and its potential impact on drinking behavior and fluid take was not the primary purpose of the study, future research should investigate its significance. Third, physical activity was not monitored prior to or during the study. Next, the sample size of the study was relatively small despite meeting the minimum number of participants based on the power calculations. Lastly, we collected spot urine samples and dietary intake only between the morning and afternoon visits instead of over a 24 h period. However, the previous literaturedemonstrated that mid- to late-afternoon spot urine samples can approximate 24 h urine concentrations [33]. Future studies should also examine the impact of thirst on these hydration indices and total water intake during extended periods. 

## 5. Conclusions

The current research determined that morning thirst had a negligible impact on later hydration status, specifically with afternoon urine indices. Furthermore, higher thirst sensation did not impact P_OSM_ or TWI-MA, although females relied slightly more on thirst sensation for TWI-MA than males. Overall, due to the complexity of thirst mechanisms, individuals may not rely solely on thirst sensation to manipulate their drinking behavior to optimize their fluid balance during their daily lives. Future research should investigate how specific thirst mechanisms and other factors could impact total water intake and the associated hydration status.

## Figures and Tables

**Table 1:** Prediction of morning thirst on afternoon hydration markers.

Afternoon Variables	Beta Coefficient	R^2^	*p*-Value
BML	−0.01	0.000	0.878
Thirst	0.22	0.074	0.021 *
U_COL_	−0.26	0.074	0.021 *
USG	−0.01	0.096	0.008 *
U_OSM_	−34.24	0.056	0.045 *
P_OSM_	−0.39	0.019	0.246
TWI-MA	70.31	0.036	0.109

BML, body mass loss; U_COL_, urine color; USG, urine specific gravity; U_OSM_, urine osmolality; P_OSM_, plasma osmolality; TWI-MA, total water intake. * Indicates statistical significance (*p* < 0.05).

**Table 2:** The relationship between morning and afternoon hydration indices for all participants.

Morning Variables	Afternoon Variables						
	U_OSM_	USG	U_COL_	BML	BM	P_OSM_	TWI-MA
U_OSM_	r = 0.387 *	r = 0.378 *	r = 0.311 *	r = 0.115	r = 0.176	r = 0.325 *	r = −0.106
USG	r = 0.360 *	r = 0.418 *	r = 0.257 *	r = 0.094	r = 0.088	r = 0.237 *	r = −0.181
U_COL_	r = 0.281 *	r = 0.280 *	r = 0.271 *	r = 0.102	r = 0.208	r = 0.184	r = −0.231
BML	r = 0.379 *	r = 0.38 *	r = 0.368 *	r = 0.792 *	r = −0.085	r = 0.100	r = −0.071
BM	r = 0.209	r = 0.188	r = 0.167	r = 0.088	r = 1.000 *	r = 0.259 *	r = 0.116
P_OSM_	r = 0.110	r = 0.148	r = 0.101	r = 0.111	r = 0.012	r = 0.188	r = 0.121

U_OSM_, urine osmolality; USG, urine specific gravity; U_COL_, urine color; BML, body mass loss; BM, body mass; P_OSM_, plasma osmolality; TWI-MA, total water intake. * Indicates statistical significance (*p* < 0.05).

**Table 3:** Comparing hydration markers between sexes (males and females) and time points (morning and afternoon).

Variables	Mean ± Standard Deviation				*p*-Value
	Morning Males	Afternoon Males	Morning Females	Afternoon Females	
BM (kg)	80.9 ± 15.2	81.1 ± 15.1	68.9 ± 14.8	69.2 ± 14.8	*p* = 0.413
BML (%)	0.34 ± 1.1	0.11 ± 1.1	−0.03 ± 0.92	−0.46 ± 1.0	*p* = 0.317
Thirst	6 ± 2	3 ± 2	5 ± 2	3 ± 2	*p* = 0.864
U_COL_	5 ± 2	4 ± 2	5 ± 2	4 ± 2	*p* = 0.742
USG	1.018 ± 0.006	1.013 ± 0.008	1.019 ± 0.009	1.014 ± 0.008	*p* = 0.738
U_OSM_ (mmol·kg^−1^)	663 ± 242	576 ± 289	717 ± 307	603 ± 301	*p* = 0.715
P_OSM_ (mmol·kg^−1^)	289 ± 5	288 ± 6	286 ± 5	284 ± 5	*p* = 0.711

BM, body mass; BML, body mass loss; U_COL_, urine color; USG, urine specific gravity; U_OSM_, urine osmolality; P_OSM_, plasma osmolality.

**Table 4:** Amount TWI-MA based on USG levels between sexes (males and females) and all participants for each time point (morning and afternoon).

Time Point	Sex	TWI-MA (mL)		
		USG < 1.020	USG ≥ 1.020	USG ≥ 1.030
Morning	Male	1536 ± 859	1520 ± 612	1275 ± 546
	Female	1382 ± 760	1631 ± 645	1061 ± 924
	All	1467 ± 809	1575 ± 618	1114 ± 813
Afternoon	Male	1601 ± 824	1365 ± 595	---
	Female	1580 ± 670	1028 ± 823	180 ± 0
	All	1590 ± 737	1237 ± 691	180 ± 0

TWI-MA, total water intake; USG, urine specific gravity.

## Data Availability

Data is not shared as other manuscripts are still in writing phase.
